# HIV Drug Resistance in Antiretroviral Treatment-Naïve Individuals in the Largest Public Hospital in Nicaragua, 2011-2015

**DOI:** 10.1371/journal.pone.0164156

**Published:** 2016-10-13

**Authors:** Santiago Avila-Ríos, Claudia García-Morales, Margarita Matías-Florentino, Daniela Tapia-Trejo, Bismarck F. Hernández-Álvarez, Sumaya E. Moreira-López, Carlos J. Quant-Durán, Guillermo Porras-Cortés, Gustavo Reyes-Terán

**Affiliations:** 1 Centre for Research in Infectious Diseases, National Institute of Respiratory Diseases, Mexico City, Mexico; 2 Hospital Dr. Roberto Calderón Gutiérrez, Managua, Nicaragua; 3 Hospital Metropolitano Vivian Pellas, Managua, Nicaragua; University of Cincinnati College of Medicine, UNITED STATES

## Abstract

**Background:**

Increasing HIV pre-treatment drug resistance (PDR) levels have been observed in regions with increasing antiretroviral treatment (ART) coverage. However, data is lacking for several low/middle-income countries. We present the first PDR survey in Nicaragua since ART introduction in the country in 2003.

**Methods:**

HIV-infected, ART-naïve Nicaraguan individuals were enrolled at Roberto Calderón Hospital, the largest national HIV referral center, from 2011 to 2015. HIV *pol* sequences were obtained at a WHO-accredited laboratory in Mexico by Sanger and next generation sequencing (NGS). PDR was assessed using the WHO surveillance drug resistance mutation (SDRM) list and the Stanford HIVdb tool.

**Results:**

283 individuals were enrolled in the study. The overall PDR prevalence based on the list of SDRMs was 13.4%. Using the Stanford HIVdb tool, overall PDR reached 19.4%; with both nucleoside and non-nucleoside reverse transcriptase inhibitor (NRTI and NNRTI) PDR levels independently reaching moderate levels (6.7% and 11.3% respectively). Protease inhibitor PDR was low (2.8%). Using NGS with 2% threshold to detect SDRMs, PDR increased to 25.3%. K103N and M41L were the most frequent SDRMs and were present mostly in proportions >20% in each individual. A significant temporal increase in NNRTI PDR was observed (p = 0.0422), with no apparent trends for other drug classes. Importantly, PDR to zidovudine + lamivudine + efavirenz and tenofovir + emtricitabine + efavirenz, the most widely used first-line regimens in Nicaragua, reached 14.6% and 10.4% respectively in 2015. Of note, a higher proportion of females was observed among individuals with PDR compared to individuals without PDR (OR 14.2; 95% CI: 7.1–28.4; p<0.0001).

**Conclusions:**

Overall PDR in the Nicaraguan cohort was high (19.4%), with a clear increasing temporal trend in NNRTI PDR. Current HIVDR to the most frequently used first-line ART regimens in Nicaragua reached levels >10%. These observations are worrisome and need to be evidenced to strengthen the national HIV program. Also, our observations warrant further nationally representative HIVDR surveillance studies and encourage other countries to perform national surveys. Cost-effectiveness studies are suggested to analyze the feasibility of implementation of baseline HIV genotyping as well as to review the choice of first-line ART regimens in Nicaragua.

## Introduction

Increasing evidence strongly suggests that the widespread use of antiretroviral treatment (ART) is resulting in increasing prevalence of circulating HIV bearing transmitted drug resistance (TDR) mutations [1, 2]. Indeed, increasing pre-ART drug resistance (PDR) levels have been observed in regions with increasing ART coverage, based mainly on fixed-dose combinations consisting of two nucleoside reverse transcriptase inhibitors (NRTI) plus a non-nucleoside reverse transcriptase inhibitor (NNRTI), including Latin America [1]. NNRTI-based regimens have a low genetic barrier to resistance, which results in treatment failure in up to 30% patients per year in low-/middle-income countries [3]. Higher acquired DR levels (ADR), in turn, have been linked with higher PDR levels [1, 4, 5]. PDR can importantly impact the effectiveness of first-line ART at the population level with individuals with PDR having higher risk of treatment failure [6–8]. Nevertheless, PDR surveillance data in some low-/middle-income countries, especially in the Latin America and Caribbean region is notably lacking [2, 9, 10]. This is the case of Nicaragua, one of the poorest countries in the Americas, in which the prevalence and patterns of PDR are not known.

By 2014, an estimate of 10,000 persons were living with HIV in Nicaragua [11]. Although HIV burden in Nicaragua is the lowest in Central America with a national general prevalence of 0.3%, the epidemic is concentrated in higher risk groups and specific geographical areas [12, 13]. According to national data obtained in 2013, HIV prevalence among self-identified men who have sex with men (MSM) was highest at the Caribbean coast with 15.5% in Bluefields and 27.9% in Bilwi. HIV prevalence among MSM in Managua was 10.4%, and 13.6% in Masaya. Among self-identified transsexual/transgender persons, HIV prevalence was 20.6% in Managua and 9.4% in Chinandega. The highest HIV prevalence in female sex workers was observed in Masaya and Managua (3.0 and 2.3% respectively) and the overall prevalence among people who inject drugs was 2.4% [13, 14].

The number of persons under ART has increased steadily since its introduction in 2003 from 22 persons under treatment in 3 health care centers to 2,935 persons in 46 centers in 2014 [13]. Access to ART is free in Nicaragua and is facilitated by the Ministry of Public Health through health centers distributed in all the 15 departments (provinces) and two autonomous zones that compose the country, although nearly half of persons who started ART in Nicaragua in 2014 were in Managua [13]. Nevertheless, considering the continuum of HIV care in Nicaragua, only 11.5% of people living with HIV are estimated to have suppressed viral load and this is strongly associated with a high ART abandonment rate (estimated over 30%) [13]. Approximately a third of all persons under ART receive their drugs through a single national referral center: Hospital Dr. Roberto Calderón Gutiérrez in Managua [15]. This center started ART administration in 2003 with the support of the Global Fund to Fight AIDS, Tuberculosis and Malaria, and functioned as reference center until 2007, when ART administration was decentralized. At present, most patients at Hospital Roberto Calderón are enrolled by spontaneous demand or diagnosed in the emergency service and are ART-naïve. Referral of migrant ART-experienced persons, mostly from Costa Rica and the USA, occurs less frequently. On average, the hospital enrolls 250 new patients per year [16]. Most people starting on first line ART receive regimens composed by two NRTI and one NNRTI, mainly zidovudine (AZT) + (lamivudine) 3TC + efavirenz (EFV), and tenofovir (TDF) + emtricitabine (FTC) + EFV [15, 17–19]. Protease inhibitors (mainly boosted lopinavir, LPV/r) are used as second line regimens. From 878 patients on ART registered at Hospital Roberto Calderón by May 2016, 15.2% were receiving first line regimens with AZT and 56% with TDF [16].

Although HIV genotyping is recommended in the national guidelines for all persons failing first-line ART regimens, this test is not performed due to technical and financial limitations. HIV genotyping has only been possible through regional clinical studies. Baseline HIV genotyping is not available in Nicaragua.

Considering this scenario, we present the first study to describe HIV PDR in Nicaragua. Knowledge on HIV PDR is important both for therapeutic decision-making and to establish public health policies on ART.

## Methods

### Ethics Statement

This study was approved by the Ethics Committees of the National Institute of Respiratory Diseases (INER) in Mexico (E06-09), and Hospital Dr. Roberto Calderón in Managua, and was conducted according to the principles of the Declaration of Helsinki. All participants gave written informed consent before blood sample donation.

## Participants

ART-naïve individuals were enrolled using convenience sampling from August 2011 to October 2015 at Dr. Roberto Calderón Hospital in Managua. All individuals without previous ART exposure arriving to clinical care were given the option to participate. Participants donated a single blood sample, which was processed at the Centre for Research in Infectious Diseases (CIENI) of the National Institute of Respiratory Diseases (INER) in Mexico City within the following 72 h of collection. Demographic data was obtained applying a questionnaire at the time of sample donation. HIV plasma viral load determination, CD4+ T cell count, HIV incidence tests, and HIV genotyping were performed and results were sent back to Hospital Roberto Calderón for patient follow-up.

### HIV *pol* Sanger Sequencing

A fragment containing the whole HIV protease (PR) and 333 codons of the reverse transcriptase (RT) (HXB2 positions 2057–3583) was bulk sequenced from free plasma virus using a previously described in-house protocol [20]. Sequences were obtained with a 3730xl Genetic Analyzer instrument (Thermo Fisher, Waltham, MA) and assembled using the web-based automated sequence analysis tool RECall (University of British Columbia, Canada) [21]. Sequencing was performed at the CIENI, INER in Mexico City, a WHO-accredited laboratory, fulfilling procedural and infrastructure requirements for good laboratory practices and quality assurance in HIV genotyping. Analyses and sequences available in S1 Appendix.

### HIV *pol* Deep Sequencing

HIV *pol* amplicons obtained for Sanger sequencing were also deep sequenced using a MiSeq instrument (Illumina, San Diego, CA). DNA libraries were generated for the *pol* PCR products using the Nextera XT DNA Sample Preparation Kit and the Nextera XT Index Kit (Illumina), according to manufacturers’ instructions. Multiplexed runs including 96 viral libraries were performed using 500-cycle MiSeq Reagent Kits v2 (Illumina), achieving a median coverage for DRM sites after filtering reads for size and quality of 16,768x (Inter-quartile range: 13,293x, 20,373x). NGS fastq files have been deposited at the NIH Short Read Archive, accession number SRP083085.

### HIVDR Analysis from NGS Runs

DR mutation (DRM) frequency within each patient was assessed from NGS runs (fastq files) using HyDRA, a freely available, web-based automated HIVDR analysis pipeline developed by the National Microbiology Laboratory of the Public Health Agency of Canada [22]. Amino acid mutations were queried against a merged DR mutation database including the WHO list of surveillance DRM (SDRMs) [23] and the Stanford HIVDR Database [24]. A minimum threshold of 1% was used to define the presence of DR mutations. Analyses available in S1 Appendix.

### HIV PDR Prevalence Analysis

HIVDR was assessed using the HIVdb [24, 25] and Calibrated Population Resistance (CPR) [26] tools of the Stanford University HIV Drug Resistance Database. For HIVdb analyses, HIVDR was defined as the presence of a penalty score ≥15 for any antiretroviral drug. CPR and HIVdb analyses were performed both with Sanger and next generation consensus sequences at 20% threshold, obtained with the HyDRA platform.

### Recency of Infection

Recent infections (RI) were identified using a multi-assay algorithm designed to minimize false recency results, as previously described [27]. The algorithm included two serology tests: the BED HIV-1 Incidence EIA (Sedia, Portland, OR) was used for screening and the HIV-1-Lag-Avidity EIA (Sedia) was used as confirmatory test. Serology tests were carried out at CIENI, INER in Mexico City, according to manufacturer’s instructions. RI were defined as individuals with less than 1 year of diagnosis, CD4+ T cell counts >200 cells/μl, plasma viral load >400 RNA copies/ml, BED HIV-1 Incidence EIA ODn<1.0 and confirmatory HIV-1-Lag-Avidity EIA ODn<1.0. The mean seroconversion period for this algorithm is 130 days (95% CI 118–142), with a false-recency rate of 0.4%.

### Phylogenetic Analyses

Sequences were aligned using ClustalW and SDRM-associated positions were eliminated. A Maximum Likelihood tree was constructed with the General Time Reversible + I + Γ model, using MEGA 6.06, including reference sequences from the Los Alamos HIV Database [28]. The best substitution model was identified with the model selection tool in MEGA 6.06. Confidence was assessed with 1000 bootstrap repetitions. Putative transmission clusters were inferred by pairwise genetic distance comparison, using the Tamura-Nei 93 evolutionary model, as previously described [29, 30]. Linkage between two sequences was established when Tamura-Nei 93 genetic distance was ≤1.5%.

## Results

A total of 283 individuals were enrolled at Dr. Roberto Calderón reference hospital in Managua. Sanger PR-RT sequences were obtained for all the participants and NGS was performed for 257 participants. The median age was 31 years (IQR 25–40). The median viral load was 4.9 log RNA copies/mL (IQR 4.2–5.5) and the median CD4+ T cell count was 284 cells/mm^3^ (IQR 92–459) suggesting late presentation to clinical care. The majority of participants were male (80%), single (63%), with high-school level of literacy (40%). The most frequent risk factor for HIV infection was heterosexual (58%) (Table 1). The great majority of viruses were subtype B (98%), with non-B subtypes represented by BD (1.1%) and BF1 (0.4%) recombinants, and C (0.4%) subtype viruses.

**Table 1:** Demographic and clinical characteristics of the participants.

Demographic / Clinical Variable	All (n = 283)	Individuals with PDR (n = 55)^a^	Individuals without PDR (n = 228)^a^	p-value^b^
VL (log RNA copies/mL) [median (IQR]	4.9 (4.2, 5.5)	4.9 (4.2, 5.5)	4.9 (4.2, 5.5)	NS
CD4+ T cells (cells/μL) [median (IQR]	284 (92, 459)	333 (115, 564)	260 (88, 441)	NS
CD4+ T cells (%)[median (IQR]	12 (6, 20)	15 (7, 24)	12 (5, 19)	NS
Age (years) [median (IQR]	31 (25, 40)	31 (26, 38)	31 (25, 40)	NS
Gender (%)				
Male	79.5	72.7	81.1	<0.0001
Female	20.1	25.5	18.9	
Civil Status (%)				
Single	63.3	58.2	64.5	NS
Domestic Partnership	20.8	21.8	20.6	NS
Married	12.4	10.9	12.7	NS
Unknown	3.5	9.1	2.2	NS
None	2.5	1.8	2.6	NS
Literacy (%)				
Primary	29.7	32.7	28.9	NS
High School	39.9	45.5	38.6	NS
Degree/Technician	25.4	14.5	28.1	0.0396
Graduate	1.1	0.0	1.3	NS
Unknown	1.4	5.5	0.4	NS
Employment (%)				
Employed	46.6	47.3	46.5	NS
Unemployed	39.6	34.5	40.8	NS
Student	9.2	10.9	8.8	NS
Unknown	4.6	7.3	3.9	NS
HIV risk factor (%)				
Heterosexual	57.6	50.9	59.2	NS
MSM	33.9	38.2	32.9	NS
PWID	1.8	5.5	0.9	0.0521
Other	1.4	0.0	1.8	NS
Unknown	5.3	5.5	5.3	NS
Recency of Infection (%)				
Recent	31.4	40.0	29.4	NS
Long-Standing	68.6	60.0	69.3	NS
Unknown	1.1	0.0	1.3	NS
HIV subtype (%)				
B	98.2	96.4	98.7	NS
Non-B	1.8	3.6	1.3	

^a^ PDR defined with Stanford HIVdb tool as the presence of a score of 15 or more to any antiretroviral drug.

^b^ Fisher’s exact or Mann Whitney tests for individuals with vs. without PDR. PDR, pre-treatment drug resistance; VL, viral load; MSM, men who have sex with men; PWID, people who inject drugs; IQR, interquartile range; NS, non-significant (p>0.05).

### PDR Prevalence and Patterns

Considering the whole study period, and using Sanger sequencing, the overall PDR prevalence based on the WHO list of SDRMs was 13.4% (95% CI: 9.7%-18.0%) (Table 2). A non-significant increase to 19.4% (95% CI: 15.0%-24.5%) in overall PDR prevalence was observed when using the Stanford HIVdb tool (p = 0.0691). This increase was mainly NNRTI-associated due to the presence of the polymorphic mutation E138A (86% of cases) and to a lesser extent V108I (14% of cases), which are not considered in the SDRMs list. NNRTI PDR was similar to NRTI PDR when using the WHO list of SDRMs (6.4% vs. 6.7%; p = 1.0), with a non-significant increase when using the HIVdb tool (11.3% vs. 6.7%; p = 0.0773) (Table 2). PI PDR was low (2.8% and 1.4% with the HIVdb and the CPR tools respectively), as well as simultaneous PDR to two ARV drug classes (1.4% and 1.1% with the HIVdb and the CPR tools respectively, in all cases NRTI + NNRTI). Estimated PDR prevalence for all ARV drug classes obtained with NGS 20% consensus sequences both with the CPR and the HIVdb tools were similar to those obtained with Sanger sequences (p>0.05 in all cases) (Table 2).

**Table 2:** HIV pre-treatment drug resistance in Nicaragua.

	HIVdb Sanger	CPR Sanger	p-value^a^	HIVdb NGS 20%	CPR NGS 20%	p-value^b^
PDR Any ARV	19.4 (15.0, 24.5)	13.4 (9.7, 18.0)	NS	21.0 (16.2, 26.5)	14.4 (10.3, 19.3)	NS
NNRTI PDR	11.3 (7.9, 15.6)	6.4 (3.8, 9.9)	0.0533	12.8 (9.0, 17.6)	7.0 (4.2, 10.8)	0.038
NRTI PDR	6.7 (4.1, 10.3)	6.7 (4.1, 10.3)	NS	7.4 (4.5, 11.3)	7.4 (4.5, 11.3)	NS
PI PDR	2.8 (1.2, 5.5)	1.4 (0.4, 3.6)	NS	2.3 (0.9, 5.0)	1.2 (0.2, 3.4)	NS
PDR Two Drug Classes	1.4 (0.4, 3.6)	1.1 (0.2, 3.1)	NS	1.9 (0.6, 4.5)	1.2 (0.2, 3.4)	NS

HIV pre-treatment drug resistance defined with Stanford HIVdb or Calibrated Population Resistance (CPR) tools for Sanger sequences and next generation consensus sequences (NGS) at 20% threshold.

^a^ Fisher’s exact test for Sanger sequence datasets: HIVdb vs. CPR.

^b^ Fisher’s exact test for NGS 20% datasets: HIVdb vs. CPR. NS, non-significant (p>0.05).

A higher proportion of females was observed among individuals with PDR compared to individuals without PDR (OR 14.2; 95% CI: 7.1–28.4; p<0.0001) (Table 1). Also, individuals with PDR showed lower literacy levels (OR 2.3; 95% CI: 1.0–5.1; p = 0.0396) and, although not frequent in the study cohort (1.8%), individuals who inject drugs showed a tendency to have higher PDR levels (OR 6.5; 95% CI: 1.1–40.0; p = 0.0521) (Table 1).

### Low Abundance Drug Resistant Variants

**Fig 1:**
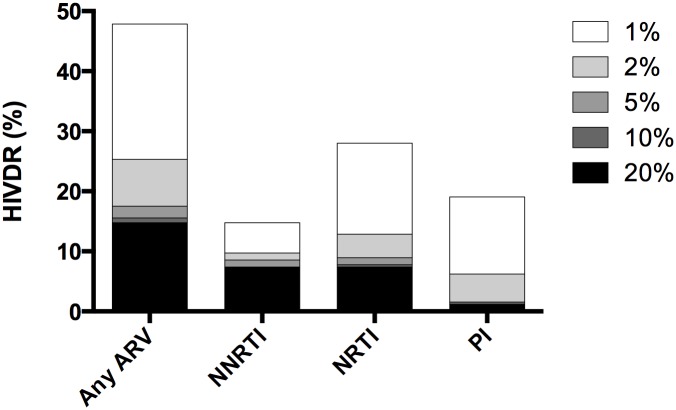
PDR levels at different sensitivity thresholds. PDR levels were estimated at 20%, 10%, 5%, 2% and 1% sensitivity thresholds using next generation sequencing as explained in Methods. Drug resistance was defined as the presence of any surveillance drug resistance mutations at the specified sensitivity threshold.

**Fig 2:**
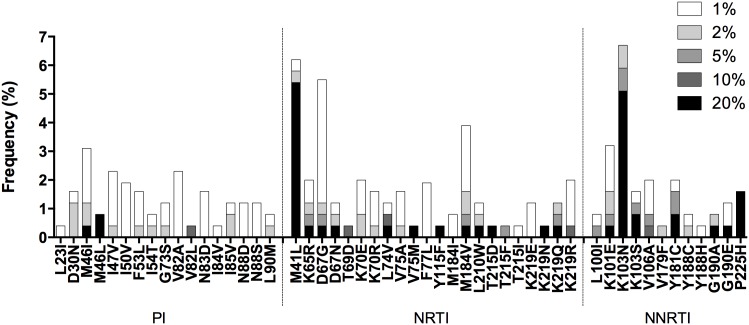
Surveillance drug resistance mutation frequency at different detection sensitivity thresholds. Drug resistance mutation frequency was determined at different thresholds using next generation sequencing as explained in Methods. Cumulative frequency for each mutation is shown. Only surveillance drug resistance mutations are shown, and are classified by drug class.

Considering only SDRMs, but lowering the mutation detection threshold to 1% using NGS, PDR to any ARV drug reached a prevalence of 47.9% (Fig 1). With a 2% threshold, PDR was 25.3%. Low-abundance variants under the 5% threshold with PI or NRTI PDR were more common than variants with NNRTI PDR (Figs 1 and 2). Taken together, low-abundance variants under the 5% threshold increased the overall PDR estimation by 30% (Fig 1).

K103N and M41L were the most frequent SDRMs in the cohort and were present mostly in proportions over 20% in each individual (5.4% and 5.1%, respectively) (Fig 2). Most PI SDRMs were present only as low-abundance variants under the 5% threshold, including L23I, D30N, I47V, I50V, F53L, I54T, G73S, V82A, N83D, I84V, I85V, N88DS, and L90M (Fig 2). Similarly, within NRTI SDRMs, K70ER, V75A, F77L, M184I, T215I, and K219E were only found under the 5% threshold, while D67G and M184V, although present in levels ≥5% in some patients, were also mostly present as low-abundance variants <5% (0.8% vs. 4.7% and 0.8% vs. 3.1%; ≥5% vs. <5% respectively).

**Fig 3:**
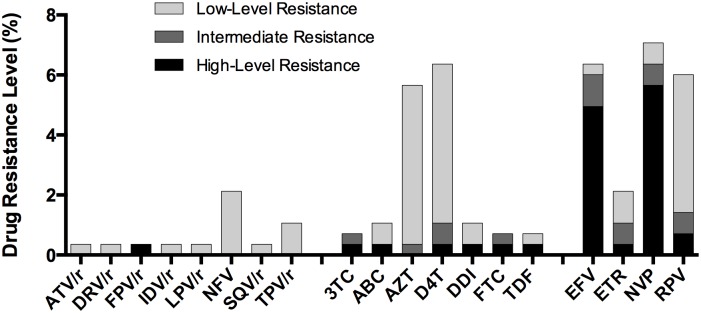
PDR levels per antiretroviral drug. PDR levels per drug were estimated using the Stanford HIVdb tool, from Sanger sequences, and classified according to the Stanford Score (SS) as high: SS≥60, intermediate: SS 30–59, or low: 15–29.

Considering only HIVDR variants >20% threshold, the most affected ARV drugs were efavirenz (EVZ), nevirapine (NVP), rilpivirine (RPV), zidovudine (AZT) and stavudine (D4T); all with PDR levels over 5% when considering at least low-level resistance (Stanford score >15) (Fig 3). EFV and NVP showed the highest levels of high/intermediate-level resistance (6.0% and 6.3% respectively) (Fig 3). Most PIs were affected by only low-level resistance at frequency under 2%.

### PDR Trends during the Study Period

**Fig 4:**
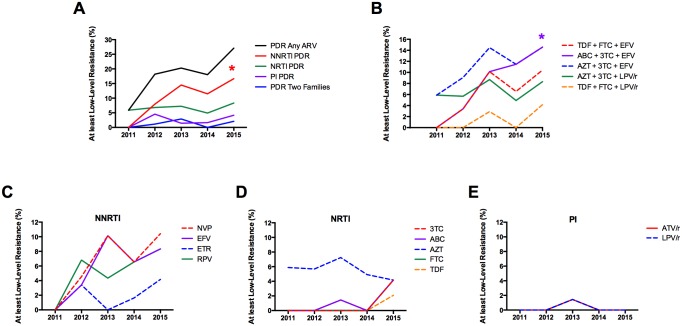
PDR temporal trends. PDR was estimated by year of enrolment using the HIVdb tool from Sanger sequences. Individuals with drug resistance were defined as those with at least low-level resistance (Stanford penalty score ≥15) to any drug of the corresponding class. A. PDR temporal trends by drug class. B. PDR temporal trends for the most widely used antiretroviral regimens in Nicaragua. C-E PDR temporal trends by drug, divided by drug class; only drugs currently used in clinical practice in the Nicaraguan context are shown. *p<0.05; linear regression, slope different to 0; the color corresponds to the significant category.

**Fig 5:**
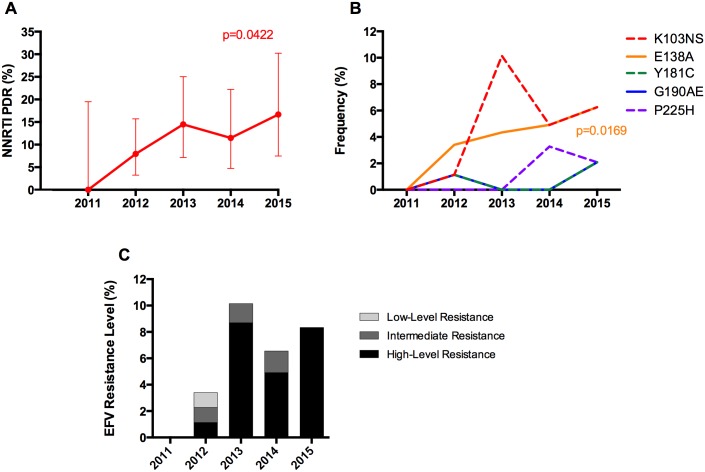
NNRTI PDR trends in Nicaragua. PDR was estimated by year of enrolment using the HIVdb tool from Sanger sequences. Individuals with drug resistance were defined as those with at least low-level resistance (Stanford penalty score ≥15) to any NNRTI. A. NNRTI PDR temporal trends; lines represent 95% confidence intervals. B. NNRTI PDR mutation frequency trends; only surveillance drug resistance mutations to NNRTI with frequency over 0.5% and E138A are included. C. PDR levels per year for efavirenz; drug resistance levels per year are classified according to the Stanford Score, as explained for Fig 3. Significant trends are shown; linear regression, slope different to 0; the color corresponds to the significant category. The number of patients enrolled by year was 17, 88, 69, 61, and 48 for 2011, 2012, 2013, 2014, and 2015 respectively.

We next analyzed PDR trends along the study period: 2011–2015. A significant NNRTI PDR increase was observed (p = 0.0422), with no apparent trends for other ARV classes (Table 3, Figs 4A and 5A). We did not observe significant increasing or decreasing PDR trends for specific ARV drugs (Fig 4C–4E), or for specific DRM frequencies except for the polymorphic mutation E138A, which showed a significant increase from 2011 to 2015 (p = 0.0169) (Fig 5B). Nevertheless, intermediate/high-level PDR to efavirenz has remained over 5% since 2013 (Fig 5C).

**Table 3:** HIV pre-treatment drug resistance by year of enrolment.

Year	n	Any ARV Drug	NNRTI PDR	NRTI PDR	PI PDR	NRTI + NNRTI PDR
2011	17	5.9 (0.1, 28.7)	0.0 (0.0, 19.5)	5.9 (0.1, 28.7)	0.0 (0.0, 19.5)	0.0 (0.0, 19.5)
2012	88	18.2 (10.8, 27.8)	8.0 (3.3, 15.7)	6.8 (2.5, 14.3)	4.5 (1.3, 11.2)	1.1 (0.0, 6.2)
2013	69	20.3 (11.6, 31.7)	14.5 (7.2, 25.0)	7.2 (2.4, 16.1)	1.4 (0.0, 7.8)	2.9 (0.4, 10.1)
2014	61	18.0 (9.4, 30.0)	11.5 (4.7, 22.2)	4.9 (1.0, 13.7)	1.6 (0.0, 8.8)	0.0 (0.0, 5.9)
2015	48	27.1 (15.3, 41.8)	16.7 (7.5, 30.2)	8.3 (2.3, 20.0)	4.2 (0.5, 14.3)	2.1 (0.1, 11.1)
p-value^a^		0.0536	0.0422	NS	NS	NS
2011–2015	283	19.4 (15.0, 24.5)	11.3 (7.9, 15.6)	6.7 (4.1, 10.3)	2.8 (1.2, 5.5)	1.4 (0.4, 3.6)

^a^ Linear regression (slope significantly different to zero) for each drug class. HIVDR defined with the HIVdb tool, using Sanger sequences. NS, non-significant (p>0.05).

Considering PDR to specific ARV drug combinations, a significant increasing temporal trend was observed for ABC + 3TC + EFV (p = 0.0044), but not for AZT + 3TC + EFV (p = 0.0727) or TDF + FTC + EFV (p = 0.0683). Importantly, PDR to AZT + 3TC + EFV and TDF + FTC + EFV, the most widely used regimens in the country, reached 14.6% and 10.4% respectively in 2015 (Fig 4B). Also noteworthy is the fact that PDR to some widely used second line regimens such as AZT + 3TC + LPV/r reached moderate levels in 2015 (8.3%) (Fig 4B).

### PDR in Recently Infected Individuals

**Fig 6:**
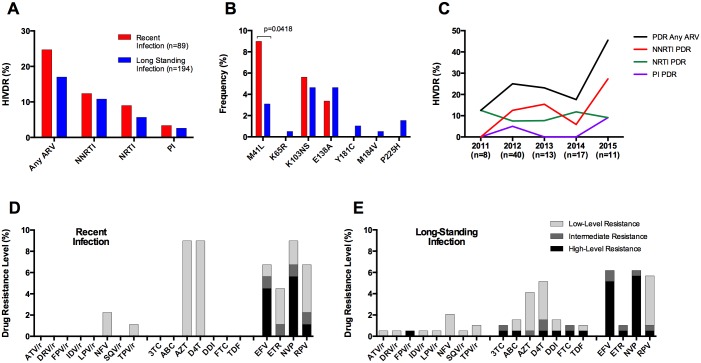
PDR in individuals with recent and long-standing HIV infection. PDR was estimated using the HIVdb tool from Sanger sequences. Individuals with drug resistance were defined as those with at least low-level resistance (Stanford penalty score ≥15) to any drug of the corresponding class. A. PDR levels by antiretroviral drug class. B. PDR levels by drug resistance mutation; relevant mutations for PDR to the most widely used ART regimens in Nicaragua are shown. C. PDR trends by year of enrolment in recently infected individuals only. D-E PDR levels by antiretroviral drug in individuals with recent and long-standing infection; drug resistance levels were classified according to the Stanford Score, as explained for Fig 3. The number of patients enrolled by year was 17, 88, 69, 61, and 48 for 2011, 2012, 2013, 2014, and 2015 respectively.

Using a multi-assay algorithm including two incidence tests, 31.4% (89/283) of individuals were estimated to present with RI (Table 1). From these, 24.7% (22/89) had PDR, with no significant difference observed when comparing with individuals with long-standing infection (17.3%, 33/191; p = 0.1495) (Fig 6A). When comparing DRM frequency in individuals with recent vs. long-standing infection, only M41L showed higher frequency in RI individuals (p = 0.0418) (Fig 6B). When assessing PDR in RI individuals by year, no significant trends during the study period were observed, although overall PDR prevalence in RI individuals in 2015 reached 45.5%, with clear NNRTI PDR dominance (27.3%) (Fig 6C). This result has to be considered with caution due to the small number of RI individuals remaining when dividing the cohort by year of enrollment. Characteristic patterns of PDR to different ARV drugs were observed when comparing recent vs. long-standing infections (Fig 6D and 6E). Regarding NRTI, RI individuals showed moderate prevalence of low-level resistance exclusively to thymidine analogs (8.9% both for AZT and D4T) and did not show PDR to 3TC, FTC, ABC, and TDF, while individuals with long-standing infection showed diverse prevalence of PDR to different NRTI, including high-/intermediate level PDR in low frequency for all NRTI (Fig 6D and 6E). PI PDR was nearly absent in RI individuals and at least low-level PDR was observed in apparently higher (but not significant) frequencies for most NNRTI drugs (Fig 6D and 6E).

### Clustering of Viruses with PDR

**Fig 7:**
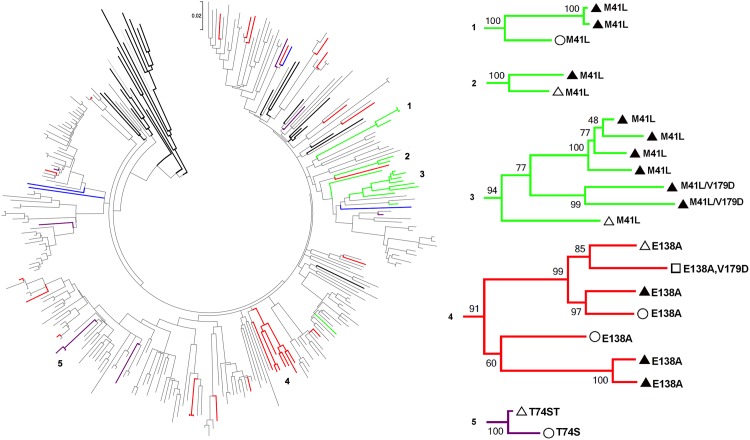
Phylogenetic relations between HIV sequences with PDR. A Maximum Likelihood tree, built with the General Time Reversible + I + Γ model, without including drug resistance positions is shown. 1000 bootstrap repetitions were used to assess confidence. Viruses with PDR to PI (purple), NRTI (green), NNRTI (red) and more than one ARV class (blue) are colored. Reference sequences were obtained from Los Alamos HIV Database (bold). Five clusters of viral sequences with PDR with bootstrap values over 90% are amplified. Circle, females; empty triangle, heterosexual males; full triangle, self-identified men who have sex with men; square, unknown risk factor.

Using pairwise Tamura-Nei 93 genetic distance ≤1.5% to establish linkage between two sequences, a total of 42 clusters were inferred (S1 Appendix). From these, 27 (64.3%) were pairs and the rest included three or more sequences, with one large cluster grouping up to 20 sequences. From all the clusters observed, five (11.9%) were composed by HIV sequences with PDR. These clusters were also evident within the phylogenetic tree and all were in branches with >90% bootstrap (Fig 7). Two large clusters of viruses with PDR were notable (n = 7 in both cases) (Fig 7). One included MSM with a singleton M41L mutation; the other combined females and males (both MSM and heterosexual) with the E138A mutation. These clusters suggest PDR transmission both heterosexually and among MSM with possible overlap between heterosexual and homosexual transmission networks by MSM who also have female partners and are not self-identified as such during the enrollment questionnaire. The presence of individuals with both recent and long-standing infection in the clusters suggests long-term stability of the M41L and E138A mutations.

### PDR in MSM and in Individuals with Heterosexual Risk of HIV Transmission

Given the observation of characteristic HIV transmission clusters, we investigated possible differences in demographic and clinical variables between MSM and individuals with heterosexual risk of HIV transmission (Table 4). Comparison of these two groups suggested two distinct HIV epidemics with individuals with heterosexual risk of HIV transmission showing lower literacy, higher unemployment and later presentation to clinical care (in all cases p<0.05, Table 4). Nevertheless, PDR rates were not statistically different between the two groups, nor the percentage of recently infected individuals.

**Table 4:** HIV PDR and demographic variables in heterosexuals and MSM.

Demographic / Clinical Variable	Heterosexuals (n = 163)	MSM (n = 96)	p-value^b^
VL (log RNA copies/mL) [median (IQR]	4.9 (4.4, 5.5)	4.8 (4.1, 5.3)	0.0496
CD4+ T cells (cells/μL) [median (IQR]	201 (70, 414)	392 (226, 517)	0.0001
CD4+ T cells (%)[median (IQR]	11 (5, 19)	16 (9, 20)	0.0139
Age (years) [median (IQR]	31 (25, 40)	30 (25, 37)	NS
Gender (%)			
Male	66.3	100.0	<0.0001
Female	33.7	0.0	
HIV PDR (%)^a^			
Any ARV drug	17.2	21.9	NS
PI	4.3	1.0	NS
NRTI	4.9	9.4	NS
NNRTI	9.2	11.5	NS
Civil Status (%)			
Single	47.9	86.5	<0.0001
Domestic Partnership	27.6	11.5	0.0028
Married	20.9	0.0	<0.0001
Unknown	3.7	2.1	
Literacy (%)			
None	4.3	0.0	0.0486
Primary	36.2	16.7	0.0010
High School	47.2	33.3	0.0369
Degree/Technician	11.7	46.9	<0.0001
Graduate	0.6	2.1	NS
Unknown	0.0	1.0	
Employment (%)			
Employed	44.2	55.2	NS
Unemployed	47.9	25.0	0.0004
Student	3.7	17.7	0.0002
Unknown	4.3	2.1	
Recency of Infection (%)			
Recent	25.2	35.4	NS
Long-Standing	73.6	63.5	
HIV subtype (%)			
B	98.2	97.9	NS
Non-B	1.2	2.1	

^a^ PDR defined with Stanford HIVdb tool as the presence of a score of 15 or more to any antiretroviral drug.

^b^ Fisher’s exact or Mann Whitney tests for heterosexuals vs. MSM. PDR, pre-treatment drug resistance; MSM, men who have sex with men; NS, non-significant (p>0.05).

## Discussion

This is the first work describing HIV PDR in Nicaragua since ART was introduced in the country in 2003. Although the survey was performed in only one HIV reference center and the observations cannot be generalized to the whole country, the fact that a third of individuals under ART in Nicaragua receive treatment at the Roberto Calderón Hospital makes this study highly valuable, providing a first national scenario on HIV PDR. Demographic and clinical data of the study cohort was consistent with national data showing that the HIV epidemic in Nicaragua affects mostly young, economically active heterosexual persons [13]. The male to female ratio was higher in the study than in the national historic cohort (4:1 vs. 1.5:1) and the proportion of persons reporting heterosexual sex as the main risk factor for HIV acquisition was lower compared to national reports (68% vs. 93%) suggesting possible enrollment biases, although it is recognized that stigmatization and discrimination most probably influence self-identification as MSM in national surveys [13]. Moreover, differences between the study population and the historic national cohort could also reflect biases of the population sectors with better access to ART, which would be better represented in the present work.

Using the Stanford HIVdb tool, the overall PDR level was high (19.4%), with both NRTI and NNRTI PDR levels independently reaching moderate levels (6.7% and 11.3% respectively). This PDR definition considers the effect of the polymorphic mutation E138A, which together with other accessory mutations increase NNRTI PDR prevalence estimations, affecting mainly RPV susceptibility. Using only SDRMs, the overall PDR level in Nicaragua was moderate (13.4%). Importantly, increasing NNRTI PDR levels were observed with time, to levels higher than 15% in 2015. Increasing NNRTI PDR trends have been reported previously for regions scaling up ART with NNRTI-based regimens [1, 2, 10]. Thus, this increasing trend in NNRTI PDR in Nicaragua is not surprising. However, the fact that NNRTI PDR reached 16.7% in 2015 is a concern and has implications for public health policy making. Of note, when comparing participants enrolled in 2011–2013 (n = 174) vs. 2014–2015 (n = 109), no significant differences in the proportion of females (21.8% vs. 17.4%), MSM (29.9% vs. 40.4%), heterosexuals (60.3% vs. 53.2%) or RI (34.5% vs. 25.7%) were observed (p>0.05 in all cases) excluding significant enrollment biases along the study period. Although it is true that part of the increasing trend in PDR was due to an increasing trend observed in E138A frequency (Fig 5), affecting RPV and ETR (drugs not used in Nicaragua), PDR to the two most common first-line ART regimens in Nicaragua reached important levels in 2015: 14.6% to AZT + 3TC + EFV and 10.4% to TDF + FTC + EFV. In particular, the use of AZT + 3TC + EFV should be revised in the Nicaraguan context. Moreover, the use of some second line regimens such as AZT + 3TC + LPV/r may also be compromised, with PDR reaching 8.3% in 2015. The most common SDRMs responsible for this PDR prevalence were K103N and M41L. This observation is relevant to the possible implementation of more affordable pre-ART HIVDR testing based on specific DRMs, compared to HIV sequencing. Indeed, viruses with PDR sequenced from RI persons included mainly one of three DRMs: M41L, K103N, or E138A. It is important to mention that even when the algorithm used to determine RI might be considered redundant, its associated false recency rate is low.

Interestingly, a large cluster of MSM with a singleton M41L mutation was identified. A similar phenomenon has been previously observed in a cohort of Swedish MSM [31]. Previous work supports the stability and lack of reversion of this mutation [32–34], as well as long-term circulation of some TAMs, including M41L, without the presence of low-abundance variants contributing with more extensive resistance [32]. In this work, we found the M41L mutation in 16 viruses sequenced with NGS; 10 of them were included in transmission clusters and 14 had the mutation in proportions over 90%, while two presented it as a low-abundance variant under the 5% threshold. Only four of the patients with M41L (all with long-standing infection) also presented other low-abundance DR variants (<5%): K103N, Y181C, K65R, and PR G73S. These observations support the long-term circulation of M41L, as well as the possibility of transmission from individuals with PDR rather than from individuals with ADR directly. The fact that M41L was more frequent in RI persons could be thus explained mostly by founder effects and MSM transmission networks, more than by acquisition of viruses with ADR and later reversion of other mutations.

Also of interest, a large cluster of patients with the E138A mutation was observed, including both females and males (MSM and heterosexual). The higher prevalence of this resistance mutation to rilpivirine in specific geographic areas, including Latin America has been previously observed [35–41]. In the present study, E138A frequency was 4.2% using Sanger sequencing. Using NGS, a total of 13 patients presented the E138A mutation over the 20% threshold and 10 additional patients presented E138 mutants as low-abundance variants <20%, including E138A, E138K and E138G. In most cases, the presence of E138A was not associated with other low-abundance DR variants. In only two cases, this mutation was accompanied by other DR variants within 5%-10% frequency: K101E and K219Q. In all, these observations suggest long-term stability of E138A, and that E138A spread may be associated to founder effects rather than increasing transmission from persons with ADR. Although the clinical role of this mutation in the context of potent ART regimens remains uncertain [42], the recommendation of ART regimens including RPV in the future in the Nicaraguan setting should be taken with caution.

Importantly, overall PDR was significantly higher in females than in males and individuals with PDR showed lower literacy levels. These observations warrant public health action when designing prevention programmes and identifies women with lower socio-economic status and their male partners as possible candidates for baseline DR testing. Also, individuals who inject drugs need to be considered as a potential vulnerable group showing higher PDR levels. When dividing persons with heterosexual risk of HIV transmission and MSM, two distinct scenarios suggesting two distinct HIV epidemics in the country were observed. Although PDR levels were similar in the two groups, heterosexuals included individuals with lower socio-economic status, lower literacy, higher unemployment rate and later presentation to clinical care compared to the MSM group. These observations are valuable for focusing and strengthening prevention efforts. Nevertheless, the lack of national representativeness of the present study is an important limitation and efforts are warranted to implementing surveys that can generate data with direct impact on national health policies, involving the national HIV program.

## Conclusions

The overall PDR in the Nicaraguan study cohort was high (19.4%), with a clear increasing temporal trend in NNRTI PDR. Also, current HIVDR to the most frequently used ARV regimens in Nicaragua reached levels >10%. These observations warrant further HIVDR surveillance studies with higher representativity and require discussion with public health policy makers in order to improve the effectiveness of ART in the Nicaraguan context. The implementation of baseline HIV genotyping as well as the choice of first line ART regimens should be discussed in the light of these findings, which can guide further cost-effectiveness analyses. When baseline HIVDR testing to all individuals initiating ART were not feasible due to limited resources, options such as increased viral load monitoring, detection of specific baseline HIVDR mutations or baseline HIVDR testing in specific groups such as females and persons who inject drugs should be considered.

## Supporting Information

S1 Appendix HIV sequence database and analyses. (XLS)
